# Secondary changes in sagittal alignment after instrumented surgery with and without corpectomy for pyogenic spondylodiscitis

**DOI:** 10.1016/j.bas.2026.106066

**Published:** 2026-04-28

**Authors:** B. Younes, B. Schatlo, P. Melich, D. Mielke, V. Rohde, T. Abboud

**Affiliations:** a Dept. of Neurosurgery, University Medical Center Göttingen, Robert-Koch-Straße 40, 37075, Göttingen, Germany; b Department of Spine Surgery, St. Antonius Hospital of the Augustinian Sisters, Jakobstraße 27-31, 50678, Cologne, Germany; c Department of Neurosurgery, University Hospital Augsburg, Augsburg, Germany

**Keywords:** Spondylodiscitis, Sagittal alignment, Posterior instrumentation, Vertebral body cage, 360° fusion

## Abstract

**Objective:**

Pyogenic spondylodiscitis is associated with substantial morbidity and mortality, and vertebral body destruction may lead to secondary spinal deformity. However, there is no consensus regarding the indication for 360° fusion compared with stand-alone posterior instrumentation. This study evaluated postoperative changes in sagittal alignment and compared radiological outcomes between these surgical strategies.

**Methods:**

Data from 90 patients who underwent instrumented surgery for pyogenic spondylodiscitis between 2013 and 2020 were retrospectively analyzed. The primary radiological endpoint was the Cobb angle at the last radiological follow-up (12 months), compared with preoperative and immediate postoperative measurements. Continuous variables are presented as mean ± standard deviation. Univariate and multivariable regression analyses were performed to identify factors associated with alignment changes.

**Results:**

Posterior instrumentation was performed in 65 lumbar and 25 thoracic cases; 360° fusion was performed in 9 lumbar and 11 thoracic cases. The mean clinical follow-up duration was 24 ± 3 months. In lumbar cases, mean Cobb angles were 26.4 ± 15.8° preoperatively, 28.9 ± 16° postoperatively, and 24.7 ± 14.2° at follow-up. Lordosis increased after surgery but decreased at follow-up (P = 0.034 and P = 0.001, respectively). In thoracic cases, mean Cobb angles were −12.3 ± 6.7°, −11.1 ± 7.1°, and −16.3 ± 9.1°, respectively, with no postoperative improvement and significant kyphosis progression at follow-up (P = 0.211 and P = 0.001, respectively). In lumbar cases, 360° fusion was associated with better sagittal alignment at last follow-up (p = 0.045) and remained an independent predictor in multivariable analysis (p = 0.038). In thoracic cases, the number of operated levels was associated with improved alignment (p = 0.048).

**Conclusion:**

Secondary changes in sagittal alignment may occur after surgery for pyogenic spondylodiscitis. A 360° fusion may provide greater long-term sagittal stability in the lumbar spine, while posterior instrumentation alone appears sufficient in the thoracic spine.

## Highlights

• Pyogenic spondylodiscitis can lead to secondary sagittal spinal alignment changes despite surgery.
• Postoperative sagittal alignment changes differ between thoracic and lumbar levels.
• In the lumbar spine, 360° fusion provides more stable long-term sagittal alignment.
• In the thoracic spine, posterior instrumentation alone provides comparable alignment.
• Surgical strategy should be tailored to the spinal region and patient-specific factors.

## 1 Introduction

Spondylodiscitis represents 4% of all cases of osteomyelitis and is a potentially life-threatening condition. Its incidence ranges from 0.5 to 2.2 per 100,000 inhabitants per year (Bonura et al., 2019; Hadjipavlou et al., 2000). The higher diagnostic efficacy obtained by magnetic resonance imaging (MRI), increased numbers of immunocompromised patients, and the longer life expectancy could be the cause of the increasing cases of spondylodiscitis in recent years (Aljawadi et al., 2019a; Klute et al., 2024). Up to 50% of patients already suffer from neurological deficits due to spine destruction upon admission to hospital. The mortality rate remains high, ranging from 4% to 29% (Lerner et al., 2005; Poutoglidou et al., 2021). *Staphylococcus aureus* is the most common microorganism identified, accounting for approximately 70% (Acharya et al., 2024; Chen et al., 2007). In the early phase of spondylodiscitis, conservative therapy with antibiotics, analgesics, and bed rest may be sufficient (Akbar et al., 2011). However, surgery is indicated in cases of antimicrobial therapy failure, neurologic deficits, epidural abscess, intolerable local or radiculopathy pain, and instability of the spine, assessed based on kyphotic deformity, pathologic fracture, and severe osseous destruction (Klöckner and Valencia, 2003; Kramer et al., 2025).

There are various surgical methods, such as pedicle-screw spondylodesis only and ventrodorsal approaches with 360° fusion (Bonura et al., 2019; Korovessis et al., 2008; Thavarajasingam et al., 2023; Yang et al., 2014). The ventrodorsal approaches with 360° fusion have many advantages, including the restoration and maintenance of the sagittal alignment of the spine, stabilization of the spinal column, and more complete debridement of the infected focus. Otherwise, dorsal spondylodesis alone can result in successful infection resolution, improved pain scores, and neurological outcomes. Comparing to the first procedure, it requires a shorter surgery time and is associated with a lower rate of blood loss and a shorter hospital stay (Ackshota et al., 2019; Deml et al., 2022; Klöckner and Valencia, 2003; Rutges et al., 2016; Younes et al., 2026).

Stability and postoperative alignment are crucial factors for treatment success, especially considering their relevance to post-surgery back pain and the development of adjacent segment disease. There is little data in the literature concerning secondary deformity after surgery in patients with spondylodiscitis. The aim of the current study is to assess secondary changes in sagittal alignment and critically review the differences between standalone posterior stabilization and dorsal spondylodesis with corpectomy in the lumbar and thoracic spine.

## 2 Materials and methods

### 2.1 Participants

This study is a retrospective analysis of patients treated for pyogenic spondylodiscitis at University Hospital Göttingen between 2013 and 2020. Included were those who underwent surgery combined with antibiotic therapy. Exclusions were made for patients treated conservatively, and cases lacking essential data.

### 2.2 Surgical indications and operative techniques

The choice of surgical approach was based on the extent of vertebral body destruction, spinal instability, and presence of segmental deformity.

1. 360° Fusion Surgery

A combined dorsal–ventral (360°) fusion was performed in patients with severe structural destruction of the spinal column, including one of the following criteria:

• vertebral body osteolysis >50%
• pathologic vertebral fractures
• significant segmental kyphosis
• persistent spinal instability requiring anterior column reconstruction

Patients in this group underwent a two-stage surgical procedure. First, dorsal transpedicular instrumentation was performed using navigated or robot-assisted techniques. This was followed by corpectomy and radical debridement of the infected tissue. Reconstruction of the anterior column was achieved using a distractable vertebral body replacement cage (Obelisc™, Ulrich Medical, Ulm, Germany) with an adjustable angulation of 0–15°, allowing restoration of anterior column support and correction of kyphotic deformity.

2. Posterior Instrumentation Alone (Stand-Alone Dorsal Stabilization)

Stand-alone posterior instrumentation was selected in patients without extensive vertebral body destruction, pathologic fracture, or significant kyphotic deformity, when sufficient spinal stability could be achieved through posterior fixation alone. In these cases, stabilization was performed using percutaneous posterior pedicle screw–rod instrumentation with navigation or robotic assistance. Whenever feasible, transpedicular screws were also placed in the affected vertebrae to enhance construct stability.

### 2.3 Postoperative management and follow ups

Postoperative assessments included a CT scan of the operated area to evaluate surgical outcome. All patients received a 2-week course of intravenous antibiotics, tailored based on microbial culture sensitivity or empirically in cases of negative cultures. This was followed by 4 to 6 weeks of oral antibiotics until CRP and leukocyte levels normalized. A hard brace was prescribed for 12 weeks post-surgery, after which patients were allowed unrestricted movement. Radiological follow-up using MRI took place 6 weeks after surgery. Further radiological follow-up CT scans were performed at 3 months and again at 12 months after surgery. Clinical follow-up was conducted annually thereafter.

### 2.4 Assessment of sagittal alignment

**Fig. 1:**
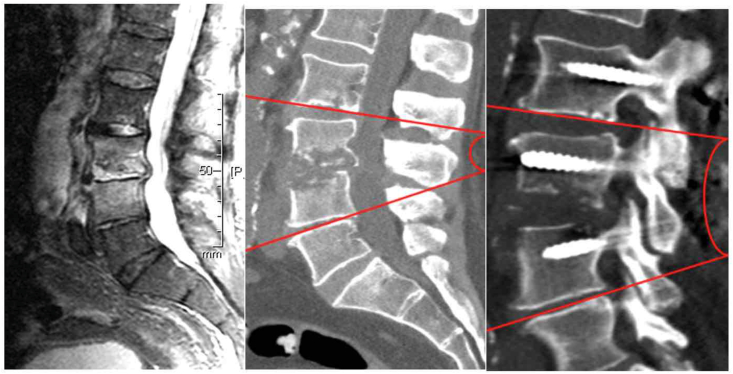
Measuring the Cobb angle before and after pedicle-screw spondylodesis surgery.

**Fig. 2:**
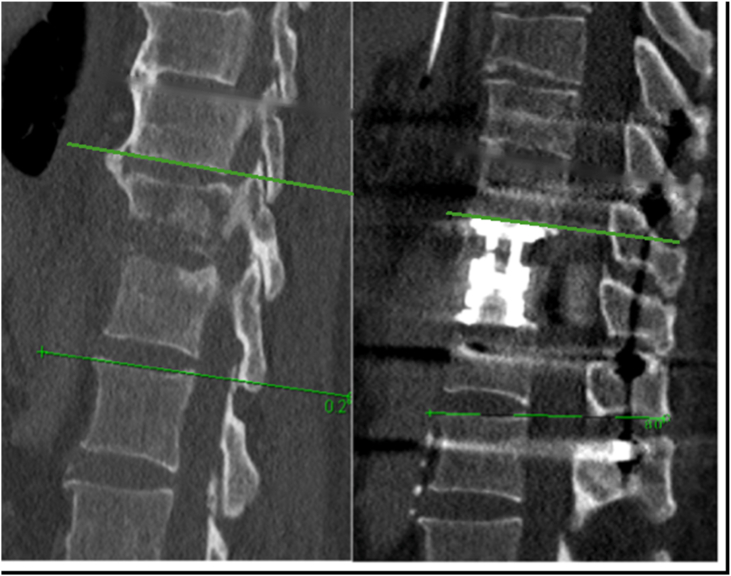
Measuring the Cobb angle before and after dorsal stabilization with ventral debridement and vertebral body replacement (360° fusion). The Cobb angle before the surgery was approximately 0°, and after surgery, it was 8°.

The primary radiological endpoint of the study was the Cobb angle at the last radiological follow-up at 12 months after surgery, which was compared with the preoperative and immediate postoperative measurements. Cobb angle was defined as the angle formed between a line drawn along the inferior endplate of the vertebra above the affected disc space and a line drawn along the superior endplate of the vertebra below the affected disc space (Fig. 1, Fig. 2). Negative values represent kyphosis, whereas positive values represent lordosis. Two specialists independently reviewed the radiographs. Discrepancies were resolved through additional review. The delta (Δ) angle, which represents the change in the Cobb angle in the lumbar and thoracic spine was then calculated for each patient at preoperative, direct postoperative and in the last radiological follow up at 12 months after surgery. Positive Δ values indicate an increase in lordosis, whereas negative Δ values indicate an increase in kyphosis.

### 2.5 Statistical analysis

For statistical analysis, patients were categorized according to the affected spinal region into two groups: lumbar and thoracic. Within each region, patients were further stratified based on the surgical approach into 360° fusion and posterior instrumentation alone groups.

Changes in Cobb angle between different time points were analyzed using a paired *t*-test. Differences in the calculated Δ Cobb angle between the two surgical groups were assessed using an independent *t*-test. Comparisons were performed across three time intervals:

• direct postoperative vs. preoperative
• last follow-up vs. direct postoperative
• last follow-up vs. Preoperative

To explore factors associated with changes in Cobb angle, univariate correlation analyses were performed between Δ Cobb (last follow-up vs. Preoperative) and the following variables: age, Charlson Comorbidity Index, obesity, osteoporosis, preoperative CRP spinal localization, number of operated levels, corpectomy and surgery duration. Variables showing a p-value <0.1 in univariate analysis were entered into multivariable linear regression models to identify independent predictors of improvement. Additional subgroup analyses were performed separately for lumbar and non-lumbar patients.

Statistical significance was defined as a p-value <0.05. All statistical analyses were performed using IBM SPSS Statistics (version 20) and Microsoft Excel (2013). Continuous variables are presented as mean ± standard deviation (SD), and categorical variables as frequencies and percentages.

### 2.6 Ethics statement

Ethical approval was secured (ethical commission of University Hospital Göttingen, application number: 3/12/17), aligning with the 1964 Declaration of Helsinki and its amendments. All procedures adhered to local and institutional laws and data protection regulations.

## 3 Results

**Fig. 3:**
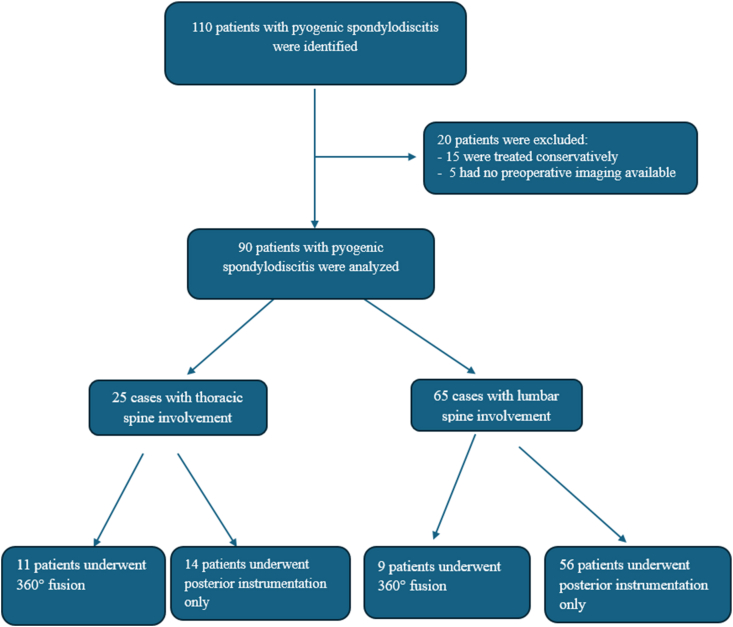
Patient flowchart showing study enrollment, exclusions and follow-up.

A total of 110 patients with pyogenic spondylodiscitis were identified. Of these, 20 were excluded: 15 were managed conservatively and 5 lacked preoperative imaging. The remaining 90 patients were included in the analysis (Fig. 3). All patients underwent surgery for pyogenic spondylodiscitis. Mean age at surgery was 66.25 ± 13.1 years. The mean duration of antibiotic treatment was 11 ± 5 weeks. Dorsal instrumentation was performed as a stand-alone procedure in 70 patients (78%) with mean age of 66.9 ± 12.3 years. Twenty patients (22%) with mean age of 64.1 ± 13.3 years underwent two-staged 360° fusion surgery.

### 3.1 Lumbar spine

The surgically treated spondylodiscitis involved the lumbar spine in 65 cases. 62% of the patients were male, and 38% were female. Among these, 9 patients (14%) underwent treatment with 360° fusion, while 56 patients (86%) underwent posterior instrumentation only. Mean Cobb angles preoperative and direct postoperatively and at last follow-up were 26.4 ± 15.8°, 28.9 ± 16° and 24.7 ± 14.2°, respectively. The lordosis increased significantly after surgery (P = 0.034) and decreased at last follow-up significantly compared to direct postoperative values, while the difference compared to preoperative values was not significant (P = 0.001 and P = 0.12, respectively).

No differences were observed between the group treated with 360° fusion and the group treated with posterior instrumentation only, regarding age (70.0 ± 12.2 vs. 66.4 ± 12.9 years, p = 0.44), preoperative CRP levels (109.7 ± 84.0 vs. 75.8 ± 49.4 mg/l, p = 0.15), surgery duration (247.5 ± 138.1 vs. 181.5 ± 76.4 min, p = 0.07) or duration of antibiotic treatment (10.5 ± 5 vs. 10 ± 2.8 weeks p = 0.46). However, patients with 360° fusion had a longer hospital stay (40.4 ± 23.4 days vs. 24.9 ± 17.4 days, p = 0.02) and a higher number of operated segments (5 ± 1 vs 2 ± 1 segments, p < 0.001). Comparisons were performed between direct postoperative and preoperative measurements, last follow-up at 12 months after surgery and direct postoperative measurements, and last follow-up and preoperative measurements. No significant differences in the Cobb angle were identified between the groups preoperatively (25.2 ± 22.4 vs. 26.5 ± 14.7, p = 0.81), direct postoperatively (28.1 ± 22.4 vs. 28.6 ± 15.0, p = 0.94), or at last follow-up (28.8 ± 20.0 vs. 24.0 ± 13.1, p = 0.35), Table 1.

**Table 1:** Characteristics of patients operated in the lumbar spine, n = number of cases.

Cobb angle	Posterior only (n = 56)	360° Fusion (n = 9)	Total (n = 65)	p
Preoperative (Degrees ±SD)	26.5 ± 14.7	25.2 ± 22.4	26.4 ± 15.8	0.81
Postoperative (Degrees ±SD)	28.6 ± 15.0	28.1 ± 22.4	28.9 ± 16.0	0.94
Follow up (Degrees ±SD)	24.0 ± 13.1	28.8 ± 20.0	24.7 ± 14.2	0.35

**Fig. 4:**
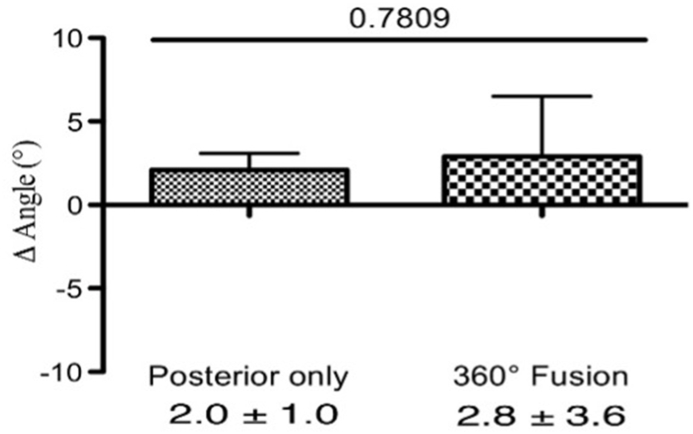
The difference in the Cobb angle in the lumbar spine (postoperative - preoperative). After pedicle-screw spondylodesis surgery the Cobb angle increased by 2.0° ± 1.0°, but after 360° fusion, it increased by 2.8° ± 3.6°.

**Fig. 5:**
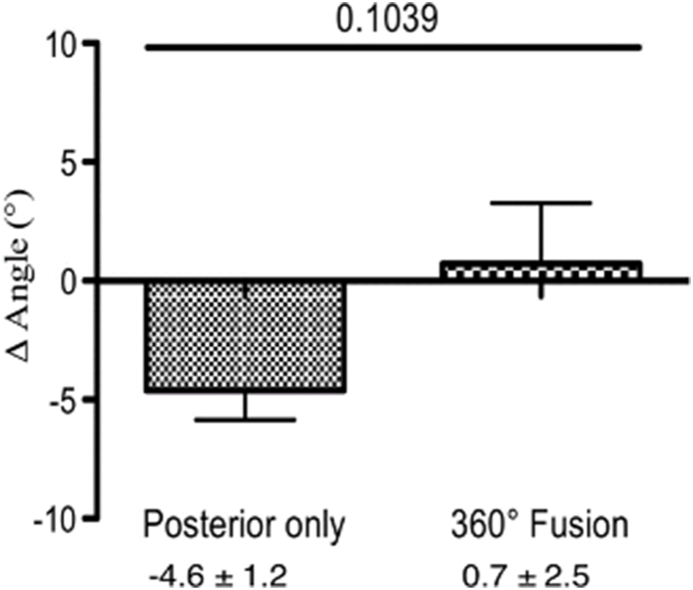
The difference in the Cobb angle in the lumbar spine (follow up - postoperative). After pedicle-screw spondylodesis surgery, the Cobb angle decreased by −4.6° ± 1.2°, but after 360° fusion, it increased by 0.7° ± 2.5°.

**Fig. 6:**
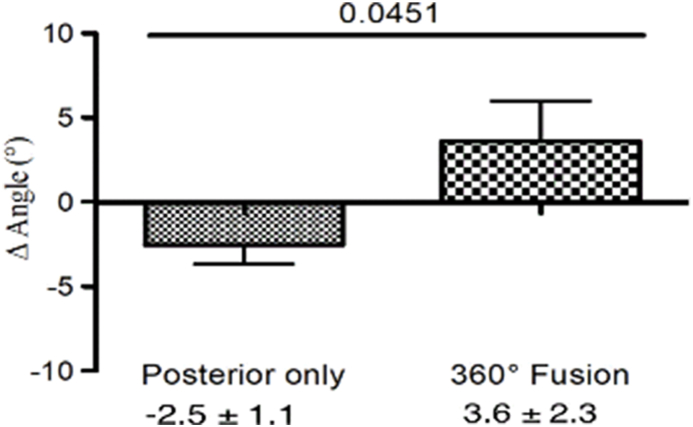
The difference in the Cobb angle in the lumbar spine (follow up – preoperative). After pedicle-screw, spondylodesis surgery the Cobb angle decreased by −2.5° ± 1.1°, but after 360° fusion, it increased by 3.6° ± 2.3°.

We calculated the Δ angle, which represents the change in the Cobb angle in the lumbar spine in both groups. No significant differences were identified between patients with 360° fusion and those with posterior instrumentation only at direct postoperative – preoperative (2.8° ± 3.6° vs. 2.0° ± 1.0°, p = 0.78, Fig. 4) or at last follow up – direct postoperative. (0.7° ± 2.5° vs. −4.6° ± 1.2°, p = 0.10, Fig. 5). However, a statistical difference was found between the groups at (last follow up - preoperative) (3.6° ± 2.3° vs. −2.5° ± 1.1°, p = 0.04, Fig. 6).

In the lumbar subgroup, univariate regression analysis showed that corpectomy was associated with greater improvement or less deterioration in the Cobb angle at follow-up compared to preoperative values (β = 6.01, p = 0.050), while obesity (β = −4.55, p = 0.097) and the Charlson Comorbidity Index (β = −1.25, p = 0.090) showed a trend toward worse outcomes. Age, osteoporosis, CRP, Surgery duration and number of operated levels were not associated with the outcome. In multivariable regression adjusted for age, corpectomy remained independently associated with greater improvement (β = 6.12, p = 0.041), whereas obesity showed a borderline association (β = −4.41, p = 0.091). Charlson Comorbidity Index and age were not significant predictors.

### 3.2 Thoracic spine

In the thoracic spine, we analyzed a total of 25 cases. 56% of the patients were male, and 44% were female. Among these, 11 (44%) patients underwent treatment with 360° fusion, while 14 (56%) patients received posterior instrumentation only. Mean Cobb angles preoperative and direct postoperatively and at last follow-up were −12.3 ± 6.7°, −11.1 ± 7.1° and −16.3 ± 9.1°, respectively. Kyphosis correction after surgery in the thoracic spine was not significant (P = 0.211) and the kyphosis increased significantly at follow-up compared to preoperative and direct postoperative values (P < 0.001 and P = 0.001, respectively). No differences were observed between the groups regarding preoperative CRP levels (100.7 ± 61.6 vs. 85.2 ± 81.3 mg/l, p = 0.66), surgery duration (230.1 ± 45.1 vs. 201.7 ± 76.4 min, p = 0.32), duration of antibiotic treatment (10.9 ± 4 vs. 10.2 ± 3 weeks, p = 0.76) or length of hospital stay (26.7 ± 9.4 vs. 27.5 ± 13.5 days, p = 0.87). However, patients in the 360° fusion group were younger (59.3 ± 12.3 vs. 68.7 ± 10.7 years, p = 0.05) and had a higher number of operated levels (5 ± 1 vs 3 ± 2, p = 0.013).

The Kyphosis was higher in the 360° fusion group preoperatively (−15.7 ± 5.7 vs. −9.6 ± 6.2, p = 0.01) and at last follow-up (−20.3 ± 7.9 vs. −13.1 ± 9.0, p = 0.04), while the difference postoperatively was not significant (−13.6 ± 7.7 vs. −9.1 ± 6.1, p = 0.11), Table 2.

**Table 2:** Characteristics of patients operated in the thoracic spine, n = number of cases.

Cobb angle	Posterior only (n = 14)	360° Fusion (n = 11)	Total (n = 25)	p
Preoperative (Degrees ±SD)	−9.6 ± 6.2	−15.7 ± 5.7	−12.3 ± 6.7	0.01*
Postoperative (Degrees ±SD)	−9.1 ± 6.1	−13.6 ± 7.7	−11.1 ± 7.1	0.11
Last Follow up (Degrees ±SD)	−13.1 ± 9.1	−20.3 ± 7.9	−16.3 ± 9.1	0.04*

**Fig. 7:**
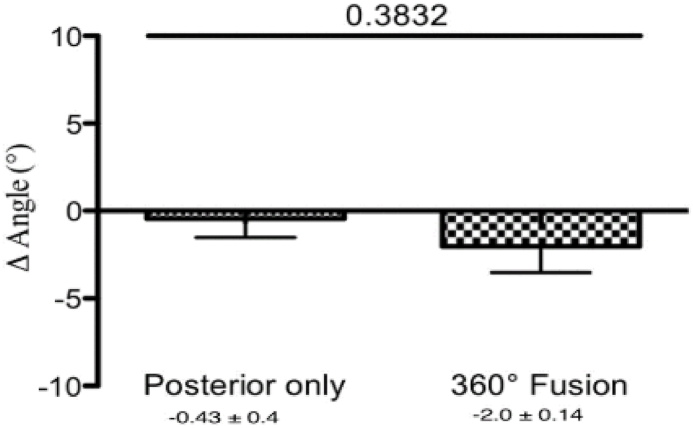
The change in the Cobb angle (Δ angle) in the thoracic spine (postoperative - preoperative). After pedicle screw spondylodesis surgery the Cobb angle decreased by −0.43° ± 0.4°, and by −2.0° ± 0.14° after 360° fusion.

**Fig. 8:**
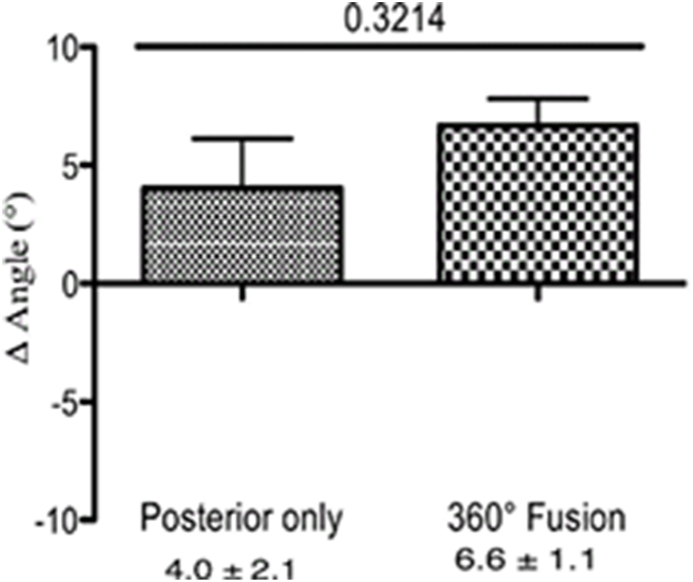
The change in the Cobb angle (Δ angle) in thoracic spine (follow up - postoperative). After pedicle screw spondylodesis surgery the Cobb angle increased by about 4.0° ± 2.1°, and by 6.6° ± 1.1° after 360° fusion.

**Fig. 9:**
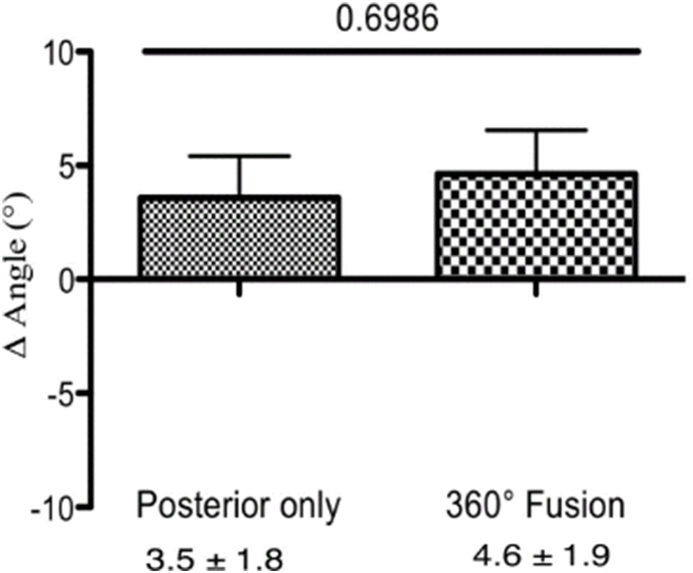
The change in the Cobb angle (Δ angle) in the thoracic spine (follow up – preoperative). After pedicle-screw spondylodesis surgery the Cobb angle increased by 3.5° ± 1.8°, and by 4.6° ± 1.9° following 360° fusion.

We calculated the Δ angle, which represents the change in the Cobb angle in the thoracic spine in both groups. No significant differences were identified between patients with 360° fusion and those with posterior instrumentation only at direct postoperative – preoperative (−2.0° ± 0.14° vs. −0.43° ± 0.4°, p = 0.38, Fig. 7), at last follow up – direct postoperative (6.6° ± 1.1° vs. 4.0° ± 2.1°, p = 0.32, Fig. 8) or at last follow up - preoperative (4.6° ± 1.9° vs. 3.5° ± 1.8°, p = 0.69, Fig. 9).

In the thoracic subgroup, univariate regression analysis showed that a higher number of operated levels was associated with greater improvement or less deterioration in the Cobb angle at follow-up compared to preoperative values (β = 1.74, p = 0.041). No significant associations were observed for age, Charlson Comorbidity Index, obesity, osteoporosis, CRP, surgery duration or corpectomy. In multivariable regression adjusted for age, the number of operated levels remained significantly associated with greater improvement or less deterioration at follow-up (β = 1.62, p = 0.048).

### 3.3 Clinical follow-up

The mean clinical follow-up period was 24 ± 3 months. Persistent infection despite prolonged antibiotic therapy occurred in 3 patients. Two of these cases required additional surgical management of the primary thoracic focus, and in one patient, methicillin-resistant *Staphylococcus aureus* (MRSA) endocarditis was identified as the primary focus. Infection relapses were observed in 10 of 90 patients (11%) during follow-up.

Six of 90 patients (7%) developed screw loosening with implant dislocation in 3 cases; four cases occurred in the lumbar spine and two in the thoracic spine. No cases of hardware breakage were observed. Revision surgery was required in 5 patients (6%). In four patients, screws were exchanged and posterior instrumentation was extended. In one patient, corpectomy with cage implantation and extension of posterior instrumentation was performed because of progressive vertebral body destruction and increasing lumbar kyphosis. Cage subsidence was observed in 2 of 20 patients (10%) who underwent anterior cage implantation, one in the thoracic spine and one in the lumbar spine. In both cases, the cages remained stable within the pedicle screw construct, and cage revision was not required.

No cases of proximal junctional kyphosis (PJK) were identified. Two patients (2%) developed adjacent segment disease in the lumbar spine requiring extension of instrumentation and decompression.

## 4 Discussion

In the current series we performed a thorough analysis of sagittal alignment in patients who underwent surgery for pyogenic spondylodiscitis and aimed at investigating the impact of type of surgical approach and surgical site on postoperative changes in the sagittal alignment in this specific patient group. For this purpose, we measured the Cobb angle in the thoracic and lumbar spine and compared the values across three time points: preoperatively, directly postoperatively, and at the last follow-up at 12 months after surgery. In addition, the ranges of direct postoperative changes in the sagittal alignment (Δ angle) were compared between patients with posterior instrumentation and 360° fusion.

We found that the Cobb angle in the lumbar spine increased significantly immediately after surgery. However, this improvement was temporary, as the Cobb angle decreased at the last follow-up (12 months) compared with the immediate postoperative values, while remaining comparable to the preoperative measurements. In contrary, surgery in the thoracic spine did not lead to an improvement of Cobb angle. Moreover, the kyphosis increased at last follow-up as compared to preoperative and direct postoperative values. Although a postoperative decrease in kyphosis followed by deterioration at the 12-month follow-up has been reported in patients with pyogenic spondylodiscitis, a differentiation based on the affected spinal segment has not been described previously (Lin et al., 2012).

### 4.1 Dorsal instrumentation alone vs. 360° fusion

Dorsal instrumentation aims to stabilize the affected spinal segments, enabling medical treatment to be more effective and allowing for early mobilization (Younes et al., 2026). Besides the debridement of the infected focus, 360° fusion can be additionally indicated to correct existing or prevent impeding spinal deformity following bony destruction (Abboud et al., 2023b). The frequency of vertebral body destruction in patients with spondylodiscitis can vary widely based on the underlying cause of the infection, the patient's health status, and the early of diagnosis and treatment. Bony destruction is associated with disturbance of sagittal alignment, which in turn is associated with decreased quality of life (Abboud et al., 2023a; Neuhoff et al., 2024; Younes et al., 2026). It also might require further surgical correction that can be unreasonable in commonly fragile patients who are known to suffer from long-term limitations in all domains of quality of life (Abboud et al., 2023a; von der Höh et al., 2025).

In our patient cohort, a significant difference was observed in the lumbar spine between patients treated with 360° fusion and those treated with posterior instrumentation alone regarding the change in Cobb angle between the preoperative measurement and the last follow-up. This finding indicates that correction of sagittal alignment after 360° fusion appears to be more stable compared with pedicle-screw spondylodesis alone. These observations are further supported by the univariate and multivariable analyses, which identified corpectomy as the only independent factor positively influencing sagittal alignment in the lumbar spine.

In contrast, no significant difference between the two surgical strategies was observed in the thoracic spine. Although patients who underwent 360° fusion were younger and had a higher number of operated levels, the correction of Cobb angles was comparable between the two groups, and the Δ angle remained similar. Moreover, both univariate and multivariable analyses demonstrated that the number of operated levels was the only factor associated with improved sagittal alignment at follow-up in the thoracic spine.

Taken together, these findings suggest that posterior spondylodesis alone may be sufficient as the first-line surgical treatment for pyogenic spondylodiscitis in the thoracic spine. In contrast, 360° fusion may be advantageous in the lumbar spine, particularly in patients who are able to tolerate the potentially higher morbidity associated with this more extensive procedure.

Transpedicular dorsal instrumentation approach covering one to two segments above and below the affected area can provide good stability and balance correction with minimal risk of injury to neurogenic and vascular structures in patients with pyogenic spondylodiscitis. Most spine surgeons have sufficient expertise in this procedure (Aljawadi et al., 2019b; Klöckner et al., 2001; Shen et al., 2021; Yang et al., 2014). Additionally, it is associated with shorter operating times and less blood loss, resulting in fewer complications compared to a 360° fusion. This could particularly beneficial in patients with a relatively osteoporotic spine (Deml et al., 2022; Klöckner and Valencia, 2003; Poutoglidou et al., 2021; Ries et al., 2015; von der Hoeh et al., 2018). In our study the operative time was numerically longer in the 360° cohort compared with posterior instrumentation alone; however, this difference did not reach statistical significance, likely due to the limited sample size of the 360° subgroup.

In recent years, many authors have suggested that 360° fusion, despite having higher complication rates, longer operating times, increased blood loss, prolonged hospital stays, and a compromise of lung function when thoracotomy is performed, is superior in infection therapy and sagittal balance restoration, resulting in fewer issues related to instrument loosening and adjacent segment disease. However, it is not recommended for patients in poor general condition (Klöckner and Valencia, 2003; von der Hoeh et al., 2018). The distractable cages enable very good correction and restoration of anterior spinal column stability, but the risk of biofilm formation on the implant surface in an infected site remains a controversial issue. The correction of sagittal alignment has been observed with both 360° fusion and posterior instrumentation alone. However, there is only a small number of published studies that have involved a thorough post-operative analysis of the sagittal profile development in the spinal region affected by spondylodiscitis (Klöckner and Wiedenhöfer, 2012; Neuhoff et al., 2025; Ries et al., 2015; Rutges et al., 2016).

Klute et al. analyzed 24 patients undergoing anterior column reconstruction of the thoracolumbar spine using expandable vertebral body replacement cages for destructive vertebral osteomyelitis and demonstrated effective restoration of local sagittal alignment. However, a significant loss of correction was observed during follow-up, highlighting the potential for long-term mechanical complications. In their series, 6 of 24 patients (25%) experienced complications, including vertebral body replacement implant dislocation (n = 1), material irritation (n = 3) and screw dislocation (n = 1), resulting in revision surgery in five cases (Klute et al., 2024). Similarly, Neuhoff et al. reported on 31 patients with a follow-up exceeding one year and found a revision rate due to implant failure of 11%, including posterior pedicle screw loosening (8%) and anterior cage subsidence (3%) (Neuhoff et al., 2024). In our cohort, the rates were comparable. Revision surgery for screw loosening and implant dislocation was required in 7% of patients, and adjacent segment disease occurred in 2%. Anterior cage subsidence was observed in 10% of cases. These findings underscore the potential for loss of surgical correction over time and reflect the inherent mechanical challenges and complexity associated with the surgical management of spondylodiscitis.

Some authors, such as Schomacher et al. and Hempelmann et al., have used procedures like posterior lumbar interbody fusion (PLIF), transforaminal lumbar interbody fusion, or extreme lateral interbody fusion in cases of spondylodiscitis without bony erosion. However, they did not observe any advantages compared to transpedicular dorsal instrumentation alone in terms of healing and sagittal alignment (Deml et al., 2022; von der Hoeh et al., 2018).

### 4.2 Clinical significance

The clinical relevance of the observed differences in Cobb angle should be interpreted with caution. Although statistically significant differences in sagittal alignment were observed between surgical techniques in the lumbar spine, there are currently no validated minimum clinically important difference (MCID) thresholds for segmental Cobb angle changes in patients with pyogenic spondylodiscitis. The concept of MCID has primarily been established for patient-reported outcome measures, such as pain scores or disability indices, rather than radiological parameters (Copay et al., 2008). Nevertheless, preservation of sagittal alignment is considered an important biomechanical factor influencing spinal stability and long-term clinical outcomes. Previous studies have demonstrated that sagittal imbalance is associated with worse pain and quality-of-life outcomes in spinal disorders (Glassman et al., 2005). Furthermore, vertebral body destruction in spinal infection may lead to progressive kyphotic deformity, which has been associated with mechanical instability and long-term functional impairment (Kramer et al., 2025; Lin et al., 2012). Therefore, although the magnitude of the angular differences observed in the present study should be interpreted cautiously, maintaining sagittal alignment remains an important surgical objective. Future prospective studies correlating radiological parameters with standardized patient-reported outcome measures are required to better determine clinically meaningful thresholds.

### 4.3 Limitations

Several limitations should be acknowledged. First, the retrospective monocentric design of the study introduces inherent sources of bias and may limit the generalizability of the findings. Second, the relatively small size of the 360° fusion subgroups, particularly in the lumbar spine, may reduce statistical power and limit the robustness of subgroup comparisons; accordingly, comparisons of complication rates between groups were not performed. In addition, a longer follow-up period may reveal further relevant information that was not captured within the current observation period. Therefore, the results should be interpreted with caution. Another limitation of the present study is the absence of standardized clinical outcome measures such as pain scores, functional disability indices, or quality-of-life assessments. Because of the retrospective design and the long study period, these parameters were not consistently documented in all patients. Therefore, the relationship between radiological sagittal alignment changes and patient-reported outcomes could not be evaluated. Future prospective studies incorporating validated clinical outcome measures are required to better determine the clinical relevance of sagittal alignment changes in pyogenic spondylodiscitis.

## 5 Conclusions

The choice of the appropriate surgical procedure is often determined on an individual basis and depends on the general health condition of the patient. Currently, no treatment has emerged as the gold standard. Following instrumented surgery for the treatment of pyogenic spondylodiscitis, secondary changes in sagittal alignment might occur in the lumbar and thoracic spine. 360° fusion might have a higher value in the lumbar spine than in the thoracic spine. Larger and perhaps prospective series are needed to validate these results.

## Ethics approval and consent to participate

Ethical approval was obtained (ethical commission of University Hospital Göttingen, application number: 3/12/17), aligning with the 1964 Declaration of Helsinki and its amendments. All procedures adhered to local and institutional laws and data protection regulations. Informed consent was not required as the study was retrospective in nature and involved the analysis of previously collected data.

## Clinical trial number

Not applicable.

## Availability of data and materials

The datasets used and/or analyzed during the current study are available from the corresponding author on reasonable request.

## Consent for publication

For this retrospective study, formal consent for publication was not required, as all data were anonymized and collected in accordance with institutional ethical standards and national data protection regulations.

## Authors' contributions

TA: designed the project, made the figure and corrected the manuscript. BY: wrote the manuscript, made the figure and analyzed data. BS: provided scientific support and corrected the manuscript. PM: made the figure, provided scientific support and corrected the manuscript. DM: provided scientific support and corrected the manuscript. VR: provided scientific support and corrected the manuscript. All authors fulfil the criteria for authorship of the International Committee of Medical Journal Editors.

## Funding

The authors declare that no funds, grants, or other financial support were received during the preparation of this manuscript.

## Conflicts of interest

The authors report no conflict of interest concerning the materials or methods used in this study or the findings specified in this paper.
